# *Burkholderia thailandensis* Is Virulent in *Drosophila melanogaster*

**DOI:** 10.1371/journal.pone.0049745

**Published:** 2012-11-27

**Authors:** Martina Pilátová, Marc S. Dionne

**Affiliations:** 1 Department of Craniofacial Development, Dental Institute, School of Medicine, King's College London, London, United Kingdom; 2 Centre for Molecular and Cellular Biology of Inflammation, Department of Immunobiology, DIIID, School of Medicine, King's College London, London, United Kingdom; Ecole Normale Supérieur de Lyon, France

## Abstract

Melioidosis is a serious infectious disease endemic to Southeast Asia and Northern Australia. This disease is caused by the Gram-negative bacterium *Burkholderia pseudomallei; Burkholderia thailandensis* is a closely-related organism known to be avirulent in humans. *B. thailandensis* has not previously been used to infect *Drosophila melanogaster*. We examined the effect of *B. thailandensis* infection on fly survival, on antimicrobial peptide expression, and on phagocytic cells. In the fruit fly, which possesses only an innate immune system, *B. thailandensis* is highly virulent, causing rapid death when injected or fed. One intriguing aspect of this infection is its temperature dependence: infected flies maintained at 25°C exhibit rapid bacterial proliferation and death in a few days, while infected animals maintained at 18°C exhibit very slow bacterial proliferation and take weeks to die; this effect is due in part to differences in immune activity of the host. Death in this infection is likely due at least in part to a secreted toxin, as injection of flies with sterile *B. thailandensis-*conditioned medium is able to kill. *B. thailandensis* infection strongly induces the expression of antimicrobial peptides, but this is insufficient to inhibit bacterial proliferation in infected flies. Finally, the function of fly phagocytes is not affected by *B. thailandensis* infection. The high virulence of *B. thailandensis* in the fly suggests the possibility that this organism is a natural pathogen of one or more invertebrates.

## Introduction

Melioidosis is a serious human and animal disease caused by the Gram-negative bacterium *Burkholderia pseudomallei*. Moist soils of rice paddies or surface water harbour this pathogen in endemic areas of Southeast Asia and Northern Australia [1]–[3]. Melioidosis can be contracted through damaged skin from *B. pseudomallei*-infected soil and water or by inhaling aerosolised bacteria [4]. In humans, melioidosis can manifest itself as a fever, mild or severe septicaemic pneumonia, skin and internal organ abscesses, and neurological conditions, such as brainstem encephalitis [3], [5]. The treatment of melioidosis is long and frequently unsuccessful; in many cases the disease recurs [6]. Currie and colleagues conducted a 10-year study of melioidosis patients and found that approximately 86% of patients who suffer septic shock as a result of this infection die [7]. The outcome of melioidosis also depends on individual circumstances and risk factors; diabetes, chronic renal disease or alcoholism have been reported to increase the rate of death in melioidosis patients [5], [8].

*B. pseudomallei* infection has been studied in Syrian golden hamsters to model melioidosis; in mice to understand various aspects of the bacterial pathogenicity, such as the effect of wild-type (WT) or mutant strains of *B. pseudomallei* on the survival of WT mice, and *in vitro* to gain insight into the intracellular life cycle of *B. pseudomallei* and its motility [9]–[11]. As this highly pathogenic bacterium is a Class B infectious agent, its study requires BSL-3 containment conditions [12]. In addition, *B. pseudomallei* is resistant to many antibiotics; restrictions on the use of antibiotics in the study of this pathogen apply [13], [14]. Due to these limitations, a safer and cheaper model for the study of some aspects of melioidosis could prove invaluable.

*B. pseudomallei* is closely related to the non-pathogenic *Burkholderia thailandensis* [15]–[17]. When discovered, *B. thailandensis* was thought to be an isolate of *B. pseudomallei*; later Brett and colleagues renamed it from *B. pseudomallei*-like to its current name [15]. Although *B. thailandensis* is mostly avirulent in mammals, high doses of *B. thailandensis* E264 kill mice [18], [19]. *B. thailandensis* and *B. pseudomallei* are motile, and live in soil and surface water, and are therefore adapted to similar environmental conditions [11], [20], [21]. Although *B. thailandensis* is not virulent in the Syrian golden hamster model [9], occasional *B. thailandensis* infections have been reported in people; in 1999 a motorcycle accident in Thailand led to melioidosis-like symptoms (here *B. thailandensis* is referred to as Ara+ *B. pseudomallei)* [22]; in the U.S., Glass and colleagues reported that *B. thailandensis* strain ATCC 700388 infection led to pneumonia and septicaemia in a 2-year old boy involved in a car accident [23].

**Figure 1:**
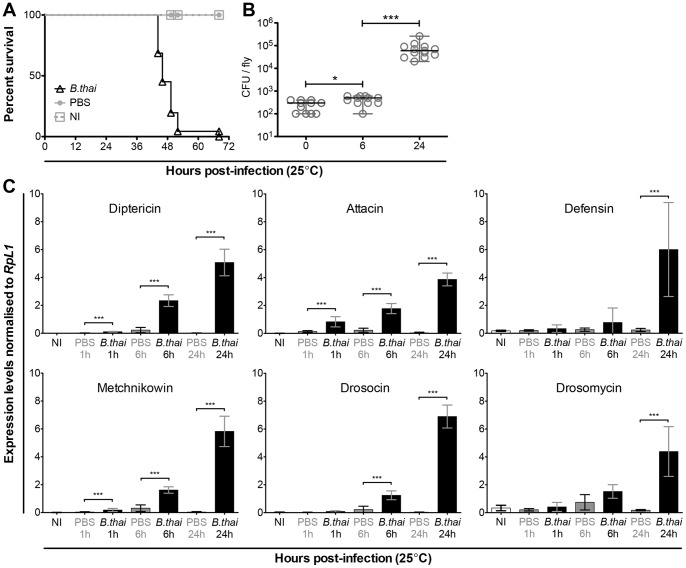
*B. thailandensis* infection kills WT male *D. melanogaster*, survives and grows in the host. (A) *Oregon-R* males were infected with WT *B. thailandensis* (*B. thai*) and died within 3.5 days of infection. Survival data was pooled from 3 independent experiments (n = min. 51 males per condition). Bacteria were injected at OD600 = 0.01, (approximately 250 CFU per fly). Mock-infected (PBS) controls were alive for the duration of this experiment. (B) *B. thailandensis* survived and multiplied inside infected flies. The data is based on 2 independent experiments (n = min. 11 males per time point). *B. thailandensis* was injected at a dose of OD600 = 0.01. Samples were collected at 0, 6 and 24 h p.i. and bacterial growth determined by plating dilutions of homogenised samples. Colonies were counted 24 h after the homogenate was plated and incubated at 37°C. Statistical significance of bacterial growth between time points was determined using Mann-Whitney test; * p<0.02 and *** p<0.0001. (C) *B. thailandensis* infection induced AMP expression in *D. melanogaster*. Three infection time points were analysed: 1, 6, and 24 h; controls were either mock-infected (PBS) or uninjected (NI). All tested AMPs were without exception significantly induced 24 h after infection. Levels of AMP mRNA were determined by qPCR. Statistical significance between levels of AMP expression was determined using Mann-Whitney test (GraphPad Prism); *** p<0.001. Data is based on 1 experiment, n = 7 males per condition; error bars represent SD.

*Drosophila melanogaster* (*D. melanogaster*) is a proven model for the study of various infections, such as *Mycobacterium marinum* [24], *Salmonella typhimurium* [25], and *Staphylococcus aureus* [26]. Despite the fact that no adaptive immunity has been discovered in *D. melanogaster*, the fly is an attractive potential model host to examine the role of innate immunity in melioidosis. The interactions of *Drosophila* with the *Burkholderia cepacia* complex have also been previously examined [27], [28]. However, to our knowledge, non-cepacia *Burkholderiaceae* have not previously been examined in *Drosophila*, despite the appeal of this organism as a potential model host to examine the role of innate immunity in melioidosis.

**Figure 2:**
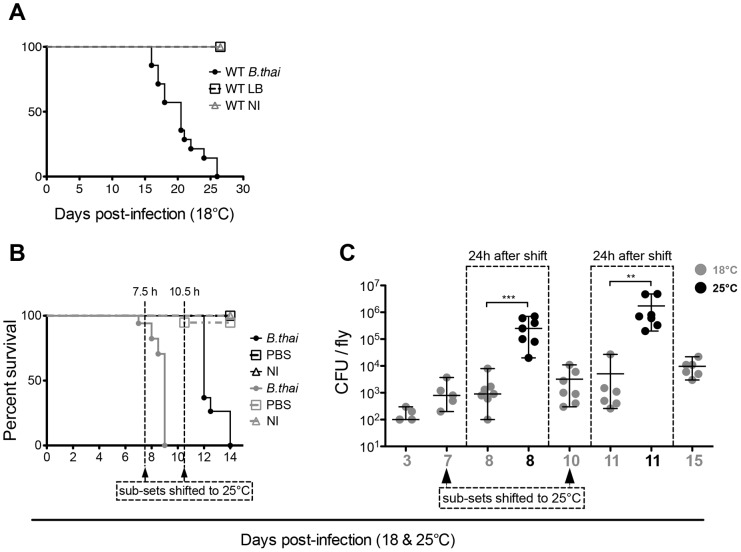
*B. thailandensis* growth at 18°C is slower than at 25°C. (A) Survival of wild-type flies infected with wild-type *B. thailandensis* E264 and kept at 18°C. (B) Infected and control flies were kept at 18°C, and subgroups were shifted to 25°C at time points 7.5 and 10.5 days after infection. Dead flies were counted twice a day. The result indicates that bacteria recovered at 25°C, and killed the flies fast. (C) *B. thailandensis* was injected at an initial dose of OD600 = 0.01. Flies were kept at 18°C (grey) and shifted to 25°C (black) at time points 7 and 10 days p.i. Subsets of equally treated flies were kept at 18°C as controls (grey). Samples were homogenised 24 h after shifting from 18 to 25°C to determine the growth of bacteria inside the flies. Samples were analysed at time points 3, 7, 8, 10, 11, and 15 days p.i. Bacterial growth was determined by plating dilutions of homogenised infected and control flies in PBS. Plated bacteria were left at 37°C for 24 h, when bacterial colonies were counted. Data is based on one experiments; n = 7 flies. Statistical significance of bacterial growth was determined using Mann-Whitney test (GraphPad Prism); ** p<0.002, *** p<0.001. Y-axis  =  log10.

The aim of this study was to evaluate *D. melanogaster* as a model organism for the study of host-pathogen interactions and the role of the innate immune response in melioidosis. The results show that *B. thailandensis* infection in *D. melanogaster* to some extent parallels *B. pseudomallei* infection in mammalian hosts. This model thus may advance our understanding of the host-pathogen interaction in terms of innate immunity.

**Figure 3:**
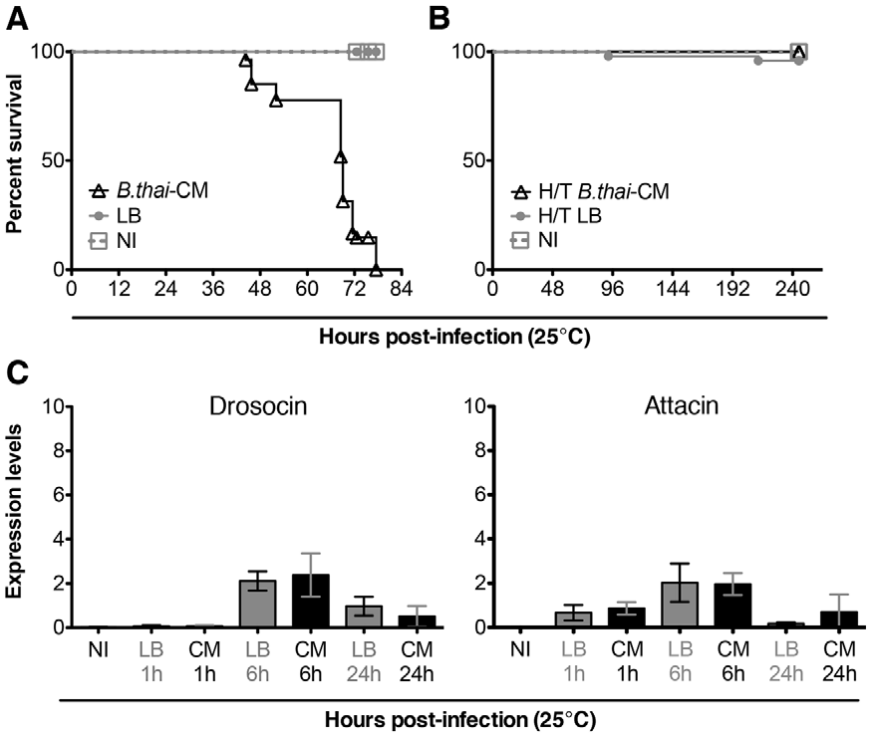
Sterile *B. thailandensis*-conditioned medium kills flies. (A) *Oregon-R* males injected with sterile-filtered *B. thailandensis*-conditioned medium (CM) died after injection. Mock-infected (LB) and uninjected (NI) controls continued to live at least for the duration of this experiment; data is based on 3 independent experiments, n = min. 56 males per condition. (B) Heat-treated conditioned medium did not kill flies; data is based on 2 independent experiments, n = min. 49 males per condition. (C) Antimicrobial peptides, Drosocin and Attacin, were not induced by *B. thailandensis*-conditioned medium. The levels of AMP mRNA were determined by qPCR; data is based on 1 experiment, n = 7 males per condition; error bars represent SD.

## Materials and Methods

### Fly stocks

To examine the effect of *B. thailandensis* infection on *Drosophila* survival, we used wild-type (WT) fly strains *Oregon-R* and *w^1118^* (DrosDel isogenic background), and a Toll and Imd pathway simultaneous loss-of-function mutant (*Dif; Rel*). Fruit flies expressing eGFP under the control of a haemocyte-specific promoter, *hemolectin* (*Hml*Δ*GAL4, UAS-2xeGFP*), were used as a control for imaging experiments to show the pattern of haemocyte distribution in the dorsal side of untreated flies (NI). All infection experiments were performed in male flies because females exhibit higher levels of nonspecific mortality due to food liquefaction.

**Figure 4:**
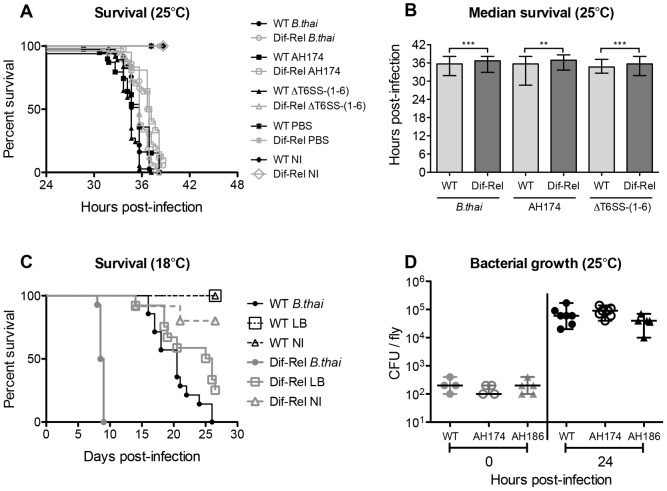
Type III (T3SS) and VI (T6SS) secretion systems are not required for virulence in *Drosophila*. (A) Survival of wild-type or *Dif; Rel* mutant flies infected with exponential-phase wild-type, T3SS-mutant (AH174) or T6SS-mutant *B. thailandensis*, maintained at 25°C, and counted at least every hour. (B) Median survival times from [A]. Statistical significance was determined using Mann-Whitney test; ** p<0.002 and *** p<0.0005. (C) Survival of wild-type and *Dif; Rel* mutant flies after infection with wild-type *B. thailandensis* E264 at 18°C. Under these conditions, *Dif; Rel* mutants were significantly shorter-lived. Statistical significance between the survival curves of infected WT and mutant flies was determined using Log-rank analysis (Mantel-Cox); p<0.0001. The data showing the WT *Drosophila* subset is the same as in [Fig. 2A]; all results shown here were obtained at the same time. (D) Proliferation of the T3SS mutant (AH174), and complemented AH186 mutant in WT *Drosophila*. Statistical significance of bacterial growth between time points was determined using Mann-Whitney test.

### Bacterial cultures

Cultures of WT *B. thailandensis* E264 (kind gift of Madeleine Moule and Brendan Wren), WT GFP-labelled and T6SS mutant *B. thailandensis* (kind gift from the Mougous lab) [29], T3SS mutant *B. thailandensis* (AH174, AH183 and the complemented strain AH186, kind gifts from the Miller lab) [18] and *Escherichia coli* DH5α were set up from frozen stocks and cultured in standard lysogeny broth (LB) at 37°C overnight with agitation. For those survival experiments indicated in the text, WT and mutant {AH174 and ΔT6SS-(1–6)} *B. thailandensis* cultures were used at an exponential-growth phase; overnight culture was diluted 1 in 10 in fresh LB and incubated for three hours at 37°C with shaking. For infection assays with phosphate buffered saline (PBS) as a control, bacterial cultures were harvested by centrifugation at 2400×g for 4 minutes at room temperature, re-suspended in PBS and calibrated using a spectrophotometer (Eppendorf); for infections with LB as a control, cultures were kept in the original growth medium and calibrated with LB to the desired density. *B. thailandensis* was calibrated to OD_600_ of 0.01, which represents approximately 250 CFU per fly when injected. *E. coli* was calibrated to OD_600_ of 1. To ensure that the LB broth was not contaminated, separate bacteria-free LB was prepared and treated in exactly the same way as LB-containing bacteria.

**Figure 5:**
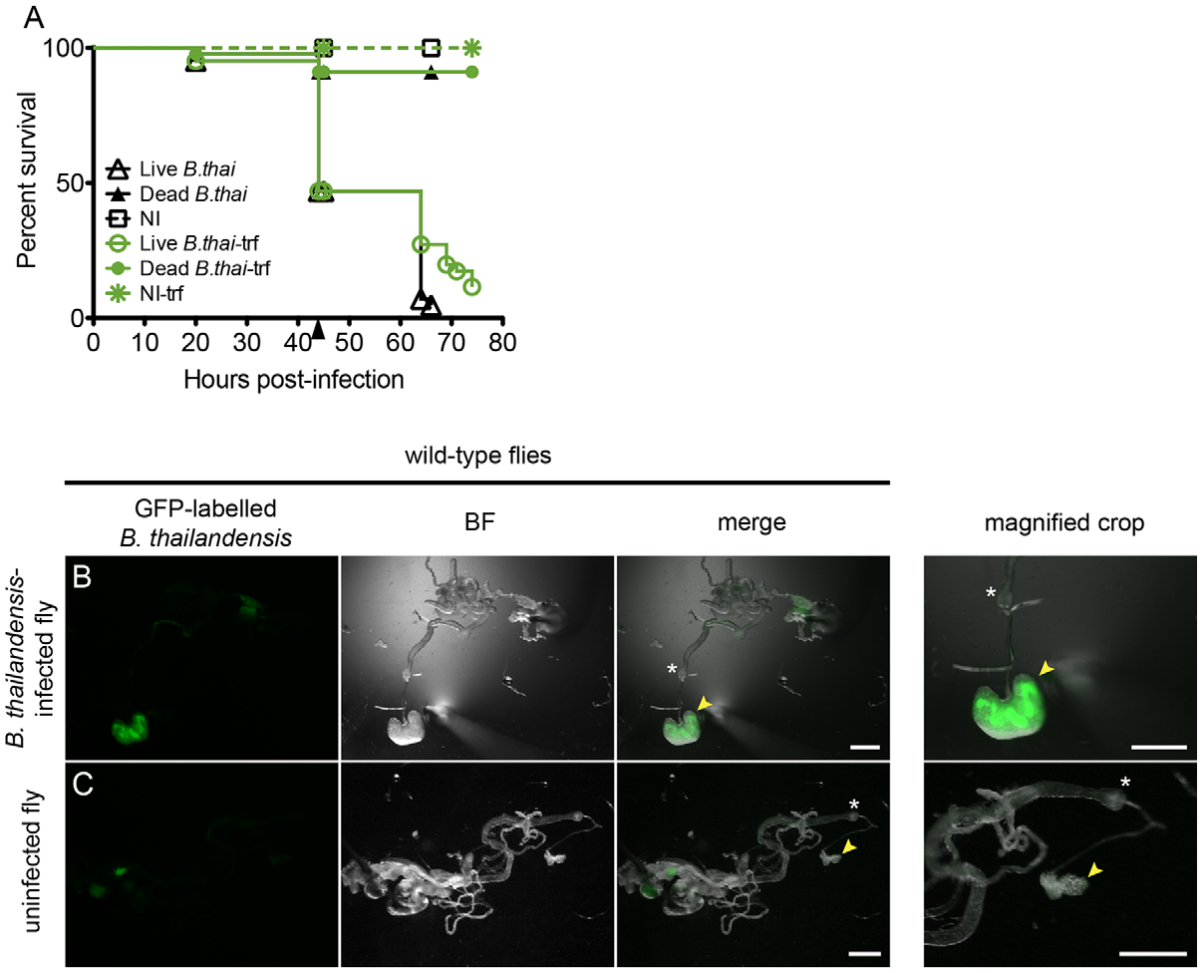
WT flies fed *B. thailandensis*-infected food are killed and have enlarged crop. (A) Survival of WT flies on infected food at 25°C. Flies kept on infected food died within 3.5 days after they were placed on this food. Second set of flies (trf) was kept on infected food and transferred to normal food, free of bacteria, at 44 hr (black arrowhead). The survival of the tranferred flies was slightly increased in comparison to the non-transferred group, but this difference was not significant. Controls were fed either food containing heat-killed *B. thailandensis* or no bacterium. The survival of the control groups was not affected. Sample size was at least 40 flies per condition. (B) Dissected gut of WT male *D. melanogaster* fed food infected with GFP-labelled *B. thailandensis*. The presence of the bacteria in the crop is confirmed by green fluorescence, which is visible only in the infected flies. (C) An uninfected control had a smaller crop. The crops of the infected and uninfected flies are shown at a higher magnification [magnified crop]. At least 3 flies were imaged per condition. Yellow arrowheads point to crop; white asterisks mark the proventriculus. Scale bars represent 500 µm.

Heat-inactivated *B. thailandensis* stock was prepared as per Sarkar-Tyson et al. [30]. The protocol was slightly modified; inactivated cultures were kept as frozen stocks at −80°C. Heat-killed *B. thailandensis* was tested for viability by incubating in liquid LB at 37°C for 48 hours (h) with shaking.

**Figure 6:**
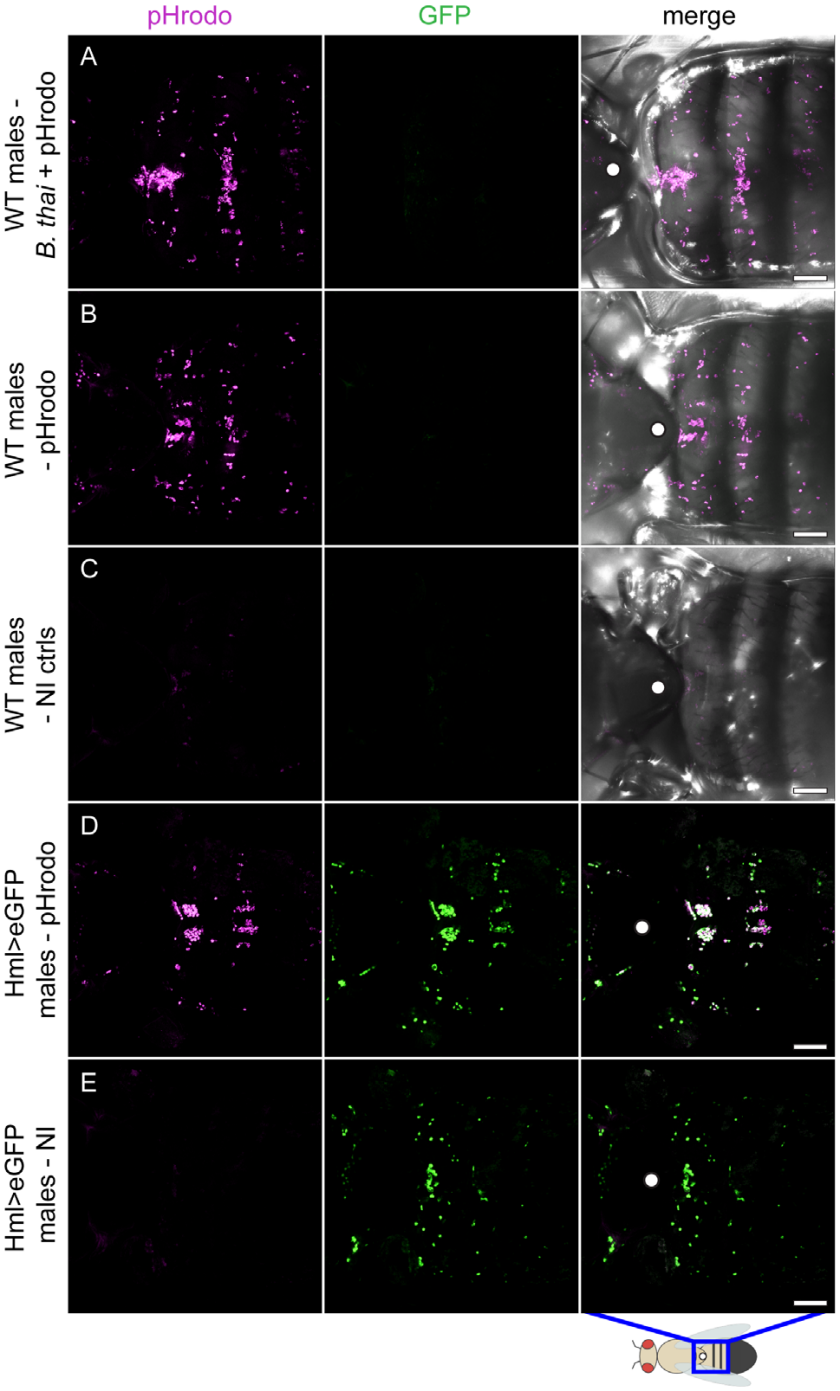
*B. thailandensis* infection does not appear to affect phagocytic function of adult plasmatocytes. (A) To examine the fate of haemocytes in this infection, WT males were infected with WT *B. thailandensis*, and 24 h later injected with pHrodo. Flies were imaged 4–5 h after pHrodo injection. The pHrodo beads were localised to haemocytes (magenta). (B) WT controls injected with pHrodo only. (C) Uninjected controls (NI). (D) pHrodo-injected flies expressing eGFP in a haemocyte-specific pattern (HmlΔGAL4, UAS-2xeGFP); at least 3 flies were imaged per condition. pHrodo is visible in magenta; co-localisation in white. (E) Untreated Hml>eGFP controls. The pattern of phagocytosed pHrodo was consistent with the pattern of haemocytes of NI flies that expressed eGFP in haemocytes. Since the pHrodo dye is bright fluorescent red only in an acidic environment, this result suggests that ∼24 h before death, haemocytes of infected flies are functioning and visible (magenta). Scale bars represent 100 µm. The cartoon shows the dorsal side of *D. melanogaster*; the blue rectangle marks the area that was imaged; the white dot marks the notum.

For experiments with *B. thailandensis*-conditioned medium (CM), overnight cultures were harvested by centrifugation, but in this case the supernatant was removed into a new tube and sterile-filtered using a 0.2 µm filter (Sartorius). To ensure that the CM contained no live bacteria, a portion of the same CM that had been injected into flies was plated on standard LB agar plate and kept at 37°C for 48 hours. As a control, 5 ml of LB was treated and processed in precisely the same way as the CM, and used for mock-infections as well as for plating.

For additional experiments, CM was heat-treated (H/T) as per modified Sarkar-Tyson et al. [30] protocol. To ensure that the H/T CM contained no live bacteria, a portion of the same H/T CM that had been injected into flies was inoculated into LB and incubated at 37°C for 24 hours. As a control, H/T LB was treated and processed in precisely the same way as the H/T CM, and used for mock-infections.

### Survival assays

Flies were kept in 30 ml tubes with roughly 8 ml *Drosophila* medium (10% brewer's yeast, 8% fructose, 2% polenta, 0.8% agar, supplemented with nipagin and propionic acid). Eclosed males of the required genotypes were collected from these tubes once a day and transferred into tubes containing fresh food. They were allowed to mature for 5–10 days prior to injection. Mature male flies were injected with a calibrated suspension of overnight bacterial culture or sterile filtered *B. thailandensis*-CM or H/T CM. Mock-infected control flies were injected with PBS, LB or H/T LB, and all injections were done using a Picospritzer® III microinjector (Intracel). In most experiments, a third set of uninjected males was kept as an untreated control. Depending on the experiment, the infected and control flies were kept at 18 or 25°C; dead flies were counted at least twice a day.

### Bacterial burden

Flies were infected as per survival assays. Infected flies were homogenised in PBS at 0, 6 and 24 h post-infection (p.i.); 0 h p.i. was the ‘input control’. One tenth of the homogenate was diluted, 1∶10, 1∶100, 1∶1000, 1∶10000, and plated on sterile LB agar plates. The plates were kept at 37°C and colonies counted 24 h after plating. Statistical significance of bacterial growth between time points was determined using Mann-Whitney test (GraphPad Prism).

### Feeding assays

Flies were maintained and selected as per survival assays. Overnight culture of *B. thailandensis* was spun at 4°C at maximum speed for 5 minutes to obtain a bacterial pellet. The spent medium was removed and the bacteria were resuspended in 1/50x PBS supplemented with 1 mM each CaCl_2_ and MgCl_2_. Fly food was prepared using dry mix containing 8.5 g fructose (Fruisana), 6.1 g dry milk powder (Marvel), 18 g Smash brand dehydrated mashed potatoes. 1 g of this dry mix was placed into each fly vial and 2 ml of bacterial suspension was added. Control food was prepared using the dry mix and PBS. The fly food was ready to use in less than 30 minutes. Experimental and control flies were put on the appropriate food and counted daily.

### mRNA extraction and cDNA synthesis

Total mRNA was extracted from infected and control flies using 100 µl of Trizol reagent (Invitrogen) as per the manufacturer's protocol. Complementary DNA (cDNA) was synthesized using the First Strand cDNA Synthesis Kit (Fermentas). The kit was used according to the manufacturer's instructions. Random Hexamers were contained in the First Strand cDNA Synthesis Kit, and used for random priming during cDNA synthesis. Obtained cDNA was analysed by quantitative RT-PCR.

### Quantitative Reverse Transcription PCR (qRT-PCR)

For quantitative analysis of *Drosophila* antimicrobial peptide gene expression, quantitative reverse transcription fluorescence PCR (qRT-PCR) was done using the double-stranded DNA dye SYBR Green (Bioline) in accordance with manufacturer's instructions. The following primer pairs were used: ***diptericin*** (*Dpt*, *CG12763*) sense, 5′-ACCGCAGTACCCACTCAATC-3′, antisense, 5′-CCCAAGTGCTGTCCATATCC-3′ ***attacin*** (*AttA*, *CG10146*) sense, 5′-CACAATGTGGTGGGTCAGG-3′, antisense, 5′-GGCACCATGACCAGCATT-3′ ***defensin*** (*Def*, *CG1385*) sense, 5′-TTCTCGTGGCTATCGCTTTT-3′, antisense, 5′-GGAGAGTAGGTCGCATGTGG-3′ ***metchnikowin*** (*Mtk*, *CG8175*) sense, 5′-TCTTGGAGCGATTTTTCTGG-3′; antisense, 5′-TCTGCCAGCACTGATGTAGC-3′ ***drosocin*** (*Dro*, *CG10816*) sense, 5′-CCATCGAGGATCACCTGACT-3′; antisense, 5′-CTTTAGGCGGGCAGAATG-3′ ***drosomycin*** (*Drs*, *CG10810*) sense, 5′-GTACTTGTTCGCCCTCTTCG-3′; antisense, 5′-CTTGCACACACGACGACAG-3′ ***ribosomal protein L4*** (*RpL1*, *CG5502*) sense, 5′-TCCACCTTGAAGAAGGGCTA-3′; antisense 5′-TTGCGGATCTCCTCAGACTT-3′.

The primer pairs were designed using Universal ProbeLibrary (Roche, https://www.roche-applied-science.com/sis/rtpcr/upl/index.jsp) to detect the desired gene transcripts, and supplied by Sigma. As a normalising gene, we used the ubiquitous *ribosomal protein L4* (*RpL1*) [31], [32]. qRT-PCR analysis was done using the Rotor-Gene 6000 (Corbett Life Science) and Rotor-Gene 6000 Series Software (Corbett Life Science).

### B. thailandensis load in infected D. melanogaster

Infected *D. melanogaster* (1 male per sample) and controls were collected and homogenised in 100 µl of PBS at required time points. One tenth of each sample was processed into a series of dilutions of 1 in 10 in PBS; 4 dilutions were made in total. 10 µl of each incremental dilution was plated on a standard LB agar plate and kept at 37°C for 24 hours. Bacterial colonies were counted on a light microscope (Nikon). Finally, to obtain the approximate numbers of viable bacteria (CFU) per fly at a given time point of infection, individual bacterial counts were multiplied appropriately, e.g. the number of colonies obtained from the first dilution (1 in 10) was multiplied by 100. Obtained results were analysed using Prism (GraphPad Software).

### Imaging

For imaging experiments, adult *Drosophila* males were treated in the same way as for survival assays, but were injected with GFP-labelled *B. thailandensis* E264 (medium dose OD_600_ of 0.1) or with dead pHrodo-conjugated *E. coli*, a rhodamine sensor of pH (pHrodo *E. coli* BioParticles®, Invitrogen). Controls were injected with PBS or uninjected (NI). Infected, injected or control flies were immobilised with the help of cyanoacrylate-based glue (Loctite), and imaged 6 or 24 h p.i. using a fluorescent (Leica) or confocal microscope (Leica TCS SP5) and capturing software (Leica Application Suite Advanced Fluorescence software). All images were processed using Adobe Photoshop CS5, and precisely the same adjustments were made to all images within an experiment.

## Results

### *B. thailandensis* E264 is pathogenic in *Drosophila melanogaster* and induces antimicrobial peptides

*Burkholderia thailandensis* E264 (*B. thailandensis*) is avirulent in people under normal conditions; however, it is highly pathogenic in wild-type (*Oregon-R*) *D. melanogaster* (5–10 days old). 100% of flies injected with *B. thailandensis* died reliably within 3.5 days of infection [Fig. 1A] and increasing bacterial dose resulted in more rapid mortality [Fig. S1A]. The survival assays were repeated several times using only the lowest bacterial dose (OD_600_ of 0.01). We also tested *w^1118^* males (DrosDel isogenic background) to see if the effect of *B. thailandensis* infection was the same as it had been in *Oregon-R* flies. The survival data is consistent in both genotypes [data not shown]. Finally, this lethality required live bacteria: heat-killed *B. thailandensis* did not cause lethality [Fig. S1B].

We next wanted to test whether the observed lethality was accompanied by bacterial proliferation. We analysed *B. thailandensis* growth in infected flies by homogenising them in PBS at 0, 6 and 24 hours p.i. and counting viable bacterial colonies. *B. thailandensis* survived in the fly; an initial phase of low growth between 0 and 6 hours after infection was followed by rapid bacterial proliferation [Fig. 1B].

As an initial test of the immune response to *B. thailandensis*, we examined induction of antimicrobial peptides (AMPs) by this infection. *B. thailandensis* strongly induced all tested AMPs, including *Diptericin*, *Attacin*, *Drosocin*, *Drosomycin, Metchinkowin,* and *Defensin* [Fig. 1C]. Despite the strong induction, bacteria proliferated and infected flies died rapidly.

*B. thailandensis* E264 is thus a highly virulent pathogen in *Drosophila*, with a low dose (∼250 CFU per fly) leading to rapid death of the host. For subsequent experiments, we have focused on the effects of the lowest verified infectious dose (OD_600_ of 0.01).

### Temperature effect on survival of infected flies, and bacterial growth

We next investigated the role of temperature in this infection. Previous experiments had shown that the distantly-related *Burkholderia cepacia* was capable of killing flies at 18°C [28]. We observed that the *B. thailandensis* infection was dramatically slowed at 18°C: median survival time increases from 2 days at 25°C [Fig. 1A] to 20 days when infected files were kept at 18°C [Fig. 2A]. This effect was qualitatively similar to, but quantitatively larger than, the temperature effect seen in *Pseudomonas aeruginosa* infection [33]. This was accompanied by a dramatic increase in bacterial doubling time. Intriguingly, flies could be infected and maintained at 18°C, with bacterial numbers stable or only very slowly increasing; when these animals were shifted to 25°C, the infection switched from chronic to acute [Fig. 2B], with bacterial numbers rapidly increasing [Fig. 2C] and causing the death of the host within one or two days of shifting to 25°C.

### Sterile *B. thailandensis*-conditioned medium is lethal to the fly

*B. pseudomallei* causes pathology in part by the production of exotoxins [34], [35]. In order to see whether some exotoxin might account for some or all of the lethality observed in this infection, we injected flies with sterile spent medium in which *B. thailandensi*s had previously grown. *B. thailandensis* was grown overnight in LB at 37°C. The culture was spun at 2400×g for 4 minutes; supernatant was removed into a new tube and sterile-filtered using a 0.2 µm filter. To ensure that the sterile conditioned medium (CM) contained no live bacteria, a portion of the same CM that was injected into flies was plated on LB agar and kept at 37°C for 48 hours; no colonies grew (data not shown). As a control for this set of experiments, LB was kept overnight at 37°C alongside the incubating *B. thailandensis* culture, processed precisely the same way as the bacterial culture, and used for mock-infections. A portion of the sterile-filtered LB was also plated to prove that it had not been contaminated; no colonies grew at 37°C in 48 hours. When the sterile-filtered *B. thailandensis*-conditioned medium was injected into WT flies, it killed them as efficiently as live *B. thailandensis* although with a delayed kinetic [Fig. 3A]. The median time to death of flies infected with an overnight culture of live *B. thailandensis* was 46 h post-infection [Fig. 1A], while with *B. thailandensis*-CM it was approximately 69 h [Fig. 3A]. In contrast, neither *E. coli*-conditioned medium nor the overnight-treated LB was able to kill flies [Fig. 3A, S1C]. Heat treatment of *B. thailandensis*-conditioned medium was sufficient to eliminate its toxicity, and heat-killed whole *B. thailandensis* had no toxic effect [Fig. 3B, S1B]. *Drosophila* injected with either *B. thailandensis* culture grown overnight in LB or with one washed and resuspended in PBS died at an approximately the same rate; survival curves were not significantly different from each other (data not shown).

Despite being lethal to WT flies, *B. thailandensis*-conditioned medium did not induce a systemic immune response: *D. melanogaster* AMPs Drosocin and Attacin, which were strongly induced by infection with live bacteria, were not induced [Fig. 3C].

### The inducible humoral immune response has differential effect on *B. thailandensis* infection depending on temperature

We had seen that infection with live *B. thailandensis* strongly stimulated antimicrobial peptide expression in the fly. As the AMP response is the most important determinant of survival in most bacterial infections in the fly, we tested the ability of flies lacking *Dif* and *Rel*, the two most prominent transcriptional effectors of this response, to survive infection with *B. thailandensis*. These animals are incapable of producing antimicrobial peptides in response to immune challenge [36], [37]. *Dif; Rel* double mutants exhibited no increase in susceptibility when infected with exponential-phase *B. thailandensis* at 25°C [Fig. 4A, 4B, Fig. S2A]. In fact, *Dif; Rel* mutants were consistently very slightly longer-lived than wild-type animals (an effect only detectable by counting dead flies at extremely frequent intervals); though this effect was consistently seen, and cannot be explained by different times of infection, its origin and importance is unclear.

Finally, we examined the interaction between environmental (temperature) effects and host genotype by infecting *Dif; Rel* mutants at 18°C. In contrast to the effect seen at 25°C, *Dif; Rel* mutants infected at 18°C died much faster than wild-type flies (median survival time  = 8 days) [Fig. 4C].

### *B. thailandensis* E264 Type III and Type VI secretion systems do not play a role in virulence to *D. melanogaster*

Having established that the humoral immune response is not critical in this infection at 25°C, we examined bacterial virulence mutants in the hope of finding some key effector of pathogenesis.

We tested the virulence of Bsa Type III secretion system mutant (AH174) and the complemented mutant (AH186); the AH174 mutant has a strong virulence defect in mice [18]. The mutation had no effect on the survival of *B. thailandensis*-infected flies at 25°C [Fig. 4A, 4B, Fig. S2B] (survival data for AH186 are not shown but were identical to both AH174 and wild-type E264). The same was true for *Dif; Rel* mutant flies. In wild-type flies, the growth of the T3SS_Bsa_ mutant was not significantly different from WT *B. thailandensis* [Fig. 4D].

We next tested the role of the Type VI secretion systems in virulence in *Drosophila*. Schwarz and colleagues observed that *B. thailandensis* lacking Type VI secretion system number 5 (ΔT6SS-5) had reduced virulence in mice, while T6SS-1 was important in *B. thailandensis* survival in competition with other Gram-negative bacteria, such as *Pseudomonas putida* and *Serratia proteamaculans* [29]. In flies, we found that, as with the Type III mutant above, a *B. thailandensis* mutant lacking all five Type VI secretion systems, ΔT6SS-(1–6), exhibited wild-type virulence at 25°C in wild-type animals and *Dif; Rel* mutants [Fig. 4A, 4B, Fig. S2C].

### Food infected with *B. thailandensis* E264 kills wild-type flies

In order to examine the effects of oral infection with *B. thailandensis*, we inoculated a potato-milk-fructose *Drosophila* food mix with the WT GFP-expressing strain, AH183 [18]. AH183 was tested in a survival assay to ensure that its virulence was similar to that of wild-type E264 [Fig. S3]. Flies transferred onto this food apparently remained healthy for at least 24 hours, but by 48 hours, 50% of the flies had died [Fig. 5A]. Flies that were surviving at this time were transferred to fresh uninfected food; these animals nonetheless succumbed to the infection. Oral infection killed flies with similar kinetics to infection by direct introduction of bacteria into the haemolymph. On dissection, GFP-expressing bacteria were clearly present in the gut [Fig. 5B, 5C]; in particular, the crop of these animals tended to be dramatically distended and often contained large amounts of GFP-positive material. No GFP-positive bacteria could be detected outside the gut in any animal at any stage of oral infection, and upon dissection the gut itself was not visibly breached by the infection.

### *D. melanogaster* haemocytes function is not impaired by *B. thailandensis*

Some infections in *Drosophila* inhibit the bactericidal phagocyte system [24], [38]–[41]. To understand what effect *B. thailandensis* has on haemocytes we used pHrodo-labelled *E. coli* BioParticles® (pHrodo). pHrodo is rhodamine-based dye that is conjugated to dead bacteria as a probe for phagocytosis; it is red fluorescent only at a low pH, such as that found in phagocytic vesicles. This fluorogenic feature allows specific imaging of phagocytosis and also, in this case, confirmed that injected pHrodo-labelled bacteria were internalised by haemocytes of *B. thailandensis*-infected *D. melanogaster* approximately 24 h before the host was killed by this infection [Fig. 6A]. The obtained data shows that the distribution of pHrodo-containing haemocytes in infected flies is comparable to that of flies injected only with pHrodo, but with no bacteria [Fig. 6B]. Untreated controls were imaged at the same time as infected flies; no fluorescence was visible, only slight auto-fluorescence was noted [Fig. 6C]. All infected and control flies were imaged in a GFP channel. In addition, we used *D. melanogaster* expressing eGFP in a haemocyte-specific manner, *Hml*Δ*GAL4, UAS-2xeGFP*, as a control to show the colocalisation of pHrodo and haemocytes [Fig. 6D]; untreated controls were also imaged [Fig. 6E]. Attempts to localize injected *B. thailandensis* using the GFP-expressing strain were stymied by inconsistent localization (data not shown).

Based on our results, *B. thailandensis* infection in *D. melanogaster* had not destroyed the phagocytic capabilities of fly haemocytes approximately 24 h before death.

## Discussion

In this study, we tested *B. thailandensis* as a potential *D. melanogaster* pathogen and found that the bacterium was highly virulent in the fly. This bacterium is mostly avirulent in humans, but exceptions have been recorded where *B. thailandensis* infection resulted in melioidosis-like symptoms [22], [23]. *Drosophila* has been shown to be a genetically tractable model in other infections [24]–[26], [42].

*B. thailandensis* survives and multiplies in infected flies. The bacterium grows well at 25–37°C [9] and when injected into *Drosophila*, it multiplies until the time of the host's death. The lethal dose of *B. thailandensis* is approximately 250 CFU per fly. Growth between 0 h and 6 h post-infection is slow and statistically insignificant; however, the bacterial burden at 24 h post-infection was significantly higher in comparison to that obtained at 6 h p.i.

Although *B. thailandensis* infection induces expression of *Drosophila* AMPs, the bacterium kills its host within 48 hours, and *Drosophila* mutants that cannot produce AMPs exhibit no increase in susceptibility to the infection at 25°C. This result suggests that *B. thailandensis* may be resistant to AMPs, much as *B. pseudomallei* is resistant to human defensin HNP-1 *in vitro* [43]. In this study *B. pseudomallei*, but not *S. typhimurium* or *E. coli*, was resistant to HNP-1 [43]. Other possible explanations for this observation include bacterial disruption of AMP production at a post-transcriptional level, or the persistence of bacteria in some sheltered compartment (for example, the phagocyte). The fact that injected *B. thailandensis* exhibited no consistent anatomical localization and did not disrupt the activity of the bactericidal phagocyte system against *E. coli* reduce the likelihood of this last possibility but do not completely preclude it.

Sterile *B. thailandensis*-conditioned medium, completely free of live bacteria, proved to be as pathogenic in the fly as live bacteria. This result suggests that *B. thailandensis* secretes an exotoxin. The exotoxin might share similarity to toxins secreted by *B. pseudomallei* [34], [35]. Although the *B. thailandensis* ‘toxin’ alone kills, the bacterial culture washed and resuspended in PBS, and thus free of the ‘toxin’, kills faster in comparison with sterile bacteria-conditioned medium, implying that the exotoxin present in spent medium cannot be the sole effector of bacterial pathogenicity. Heat-treatment eliminated the activity of this toxin, suggesting that it may be proteinaceous (and is in any case unlikely to be a stable small molecule). The identity of this toxin is of clear interest.

The mechanism of reduced virulence of the *B. thailandensis* at low temperature (18°C) is not yet clear. One possibility is that the activity of the implied exotoxin may be reduced at low temperatures; this effect has been observed previously with ricin and shiga toxin [44], [45]. In this regard, it may be relevant that, in addition to the *B. thailandensis* exotoxin for which we provide evidence here, *B. pseudomallei* produces exotoxin and proteases [34], [35].

Conversely, the observation that *Dif; Rel* mutants do exhibit significant immune compromise relative to wild-type animals at 18°C suggests that either antimicrobial peptides might be more efficient at cooler temperatures or the bacterial surface might be changed in some way at lower temperature, rendering it more sensitive to the effects of antimicrobial peptides. Speculating further, it might be possible that at 18°C *B. thailandensis*' reproduction and dynamics are slower, and any potential cellular invasion might occur at a reduced pace, thus giving the AMPs more time to be efficient. Whereas in the *Dif; Rel* mutants, the bacterial replication and dynamics might be the same as in wild-type *Drosophila*, but the absence of AMPs in the immunocompromised mutants might result in increased virulence. Finally, this effect and the temperature effect on bacterial virulence might be two sides of the same coin, with a complex interaction between specific bacterial virulence factors and relative activity levels of different immune effectors giving rise to the observed dramatic changes in infection dynamics at different temperatures.

Although neither the T3SS nor T6SS appear not to affect the function of *Drosophila* haemocytes, our observation was made only qualitatively: phagocytic index was not quantified as had been done in a previous study, in which *P. aeruginosa* T3SS was shown to interfere with haemocyte phagocytic function [38]. It remains possible that the T3SS or the T6SS mutants might exhibit a detectable change in virulence if assayed in a more sensitive fashion, such as competitive index as previously shown for closely related Bcc species [27], [29].

The fact that *B. thailandensis* persisted in the gut and ultimately killed the fly after oral infection is particularly intriguing given the recent observation that pesticide-degrading *Burkholderia* strains are specific beneficial endosymbionts of several important phytophagous insects [46]. We were unable to detect *B. thailandensis* crossing the gut barrier; that said, it is not clear whether its deleterious effects in the gut are due to toxin secretion acting on the host, nutrient effects, or undetected systemic infection.

One aim of this study was to establish whether infection of *D. melanogaster* with *Burkholderia thailandensis* could be a useful model for mammalian melioidosis. Though flies are rapidly killed by *B. thailandensis*, the fact that neither Type III nor Type VI secretion systems appear to be required for virulence in the fly suggests that many virulence factors will not be conserved in this host, potentially limiting its general utility. Nonetheless, several aspects of this infection, including the presence of an apparent heat-labile exotoxin and the ability to kill flies by feeding, and the previously-observed association of other *Burkholderaciae* with insects, represent intriguing avenues for further study.

## Supporting Information

Figure S1 **Control infections with WT or heat-killed** ***B. thailandensis*** **, or with conditioned medium.** (A) Infected *D. melanogaster* was killed in a dose-dependent manner. The result is based on a single experiment; n = 19 flies per genotype per condition. Three infectious doses were tested: OD600 = 0.01 (low), 0.1 (medium), and 1 (high). Mock-infected (PBS) and untreated (NI) controls were alive for the whole duration of this experiment. (B) Heat-killed (H/K) *B. thailandensis* was avirulent in WT males at 25°C. High dose of *B. thailandensis* was OD600 of 1; low OD600 of 0.01; n = min. 16 flies per condition. (C) *E. coli*-conditioned medium (*E.coli*-CM) was not infectious at 25°C in comparison to that of *B. thailandensis* (*B.thai*-GFP-CM); n = min. 14 flies per condition.
(TIF)

Figure S2 **Infections of WT and mutant** ***Drosophila*** **with wild-type, T3SS or T6SS** ***B. thailandensis*** **.** Survival curves isolated from [Fig. 4A] showing data of WT and *Dif; Rel* mutant *D. melanogaster* infected with (A) WT *B. thailandensis*, (B) T3SS mutant, or (C) T6SS mutant, at 25°C.
(TIF)

Figure S3 **Control infections with GFP-labelled** ***B. thailandensis*** **.** Flies infected with GFP-labelled *B. thailandensis* died within 2 days p.i., which is comparable to infections with non-GFP-labelled *B. thailandensis*; n = 20 flies per condition.
(TIF)
