# Supplementary figures and images for: Burkholderia thailandensis Is Virulent in Drosophila melanogaster
Source: PLoS One. 2012 Nov 27;7(11):e49745. doi: 10.1371/journal.pone.0049745 (PMC3507839; doi:10.1371/journal.pone.0049745)

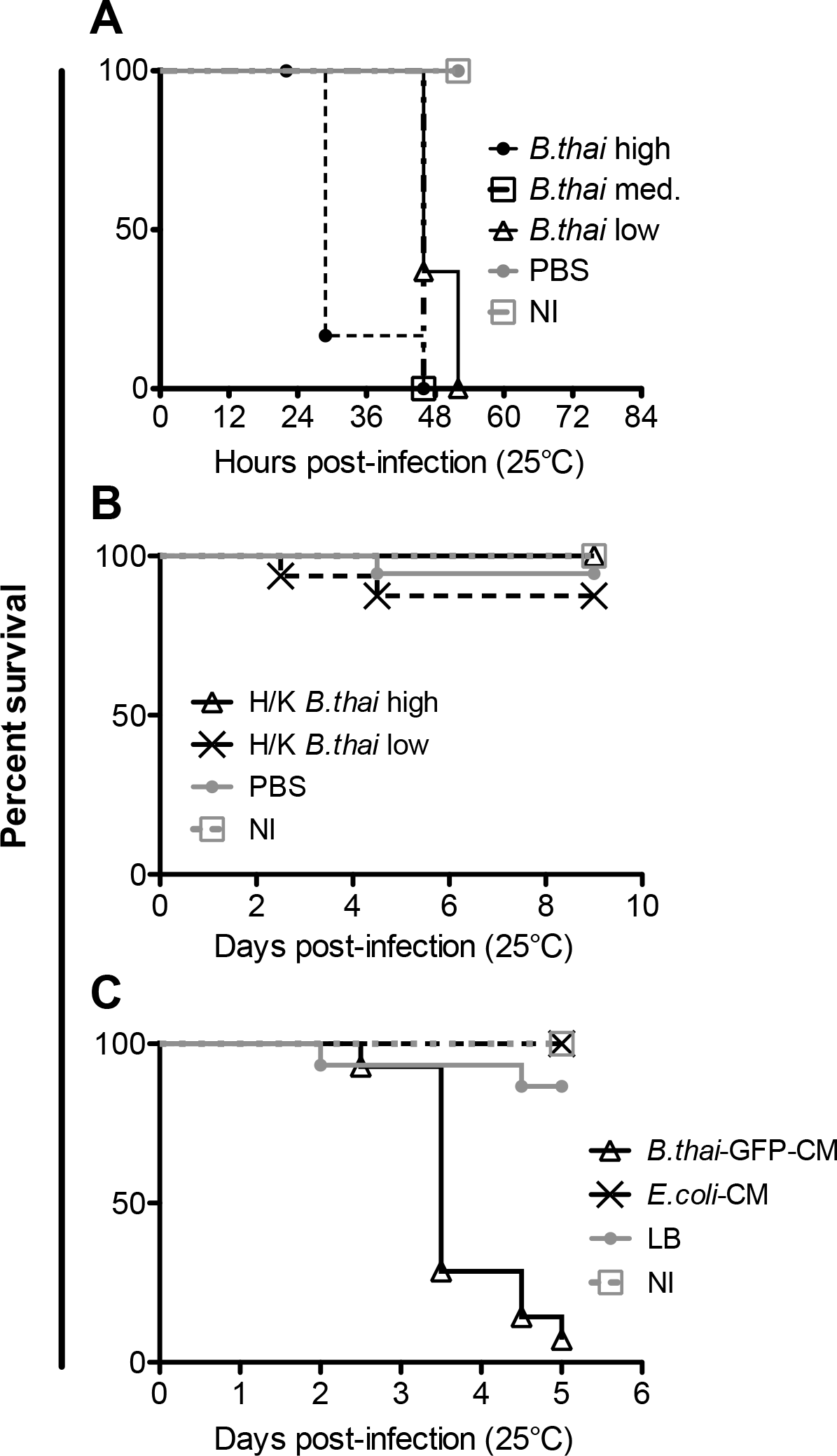

Supplement: Figure S1 — Control infections with WT or heat-killed B. thailandensis , or with conditioned medium. (A) Infected D. melanogaster was killed in a dose-dependent manner. The result is based on a single experiment; n = 19 flies per genotype per condition. Three infectious doses were tested: OD600 = 0.01 (low), 0.1 (medium), and 1 (high). Mock-infected (PBS) and untreated (NI) controls were alive for the whole duration of this experiment. (B) Heat-killed (H/K) B. thailandensis was avirulent in WT males at 25°C. High dose of B. thailandensis was OD600 of 1; low OD600 of 0.01; n = min. 16 flies per condition. (C) E. coli-conditioned medium (E.coli-CM) was not infectious at 25°C in comparison to that of B. thailandensis (B.thai-GFP-CM); n = min. 14 flies per condition. (TIF) [file pone.0049745.s001.tif]

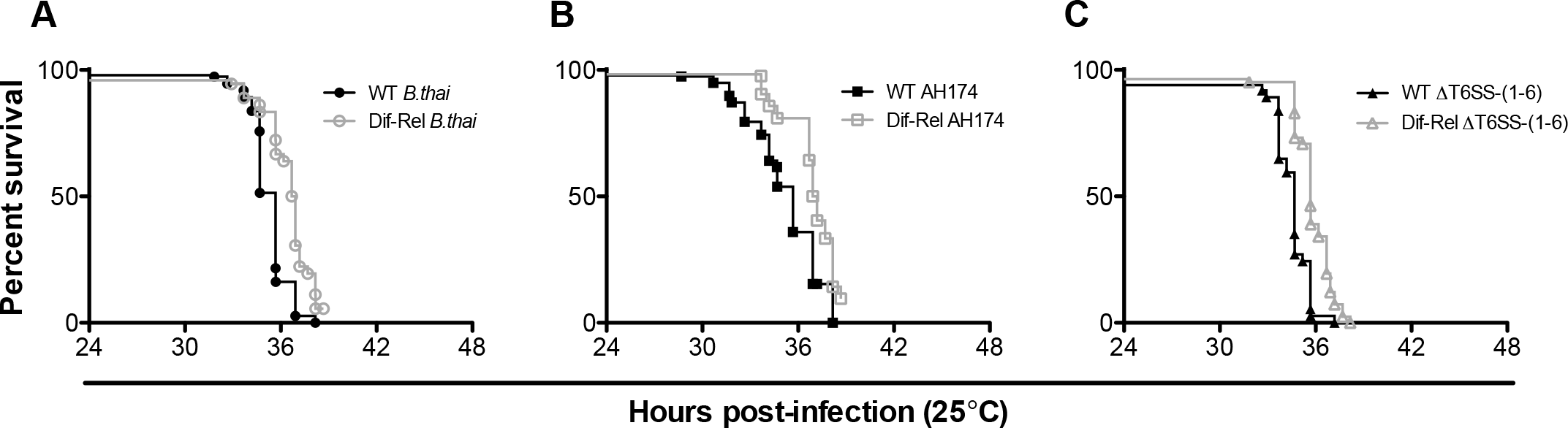

Supplement: Figure S2 — Infections of WT and mutant Drosophila with wild-type, T3SS or T6SS B. thailandensis . Survival curves isolated from [Fig. 4A] showing data of WT and Dif; Rel mutant D. melanogaster infected with (A) WT B. thailandensis, (B) T3SS mutant, or (C) T6SS mutant, at 25°C. (TIF) [file pone.0049745.s002.tif]

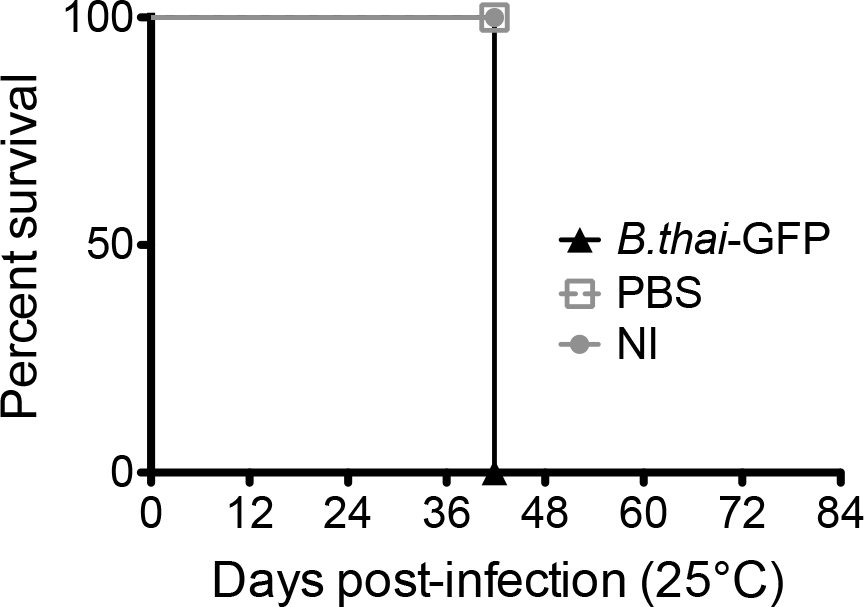

Supplement: Figure S3 — Control infections with GFP-labelled B. thailandensis . Flies infected with GFP-labelled B. thailandensis died within 2 days p.i., which is comparable to infections with non-GFP-labelled B. thailandensis; n = 20 flies per condition. (TIF) [file pone.0049745.s003.tif]
